# Challenges and future strategies for management of otomycosis caused by *Fusarium* species: A systematic review and meta-analysis

**DOI:** 10.22034/cmm.2025.345494.172

**Published:** 2025-12-17

**Authors:** Iman Haghani, Zahra Taheri Rizi, Firoozeh Kermani, Javad Javidnia, Mona Ghazanfari, Behrad Roohi, Maryam Ghafari, Mohammad Taghi Hedayati, Shaghayegh Khojasteh, Gholamreza Shokoohi, Mohsen Nosratabadi, Mahdi Abastabar, Suzana Otasevic, Zahra Farokhi, Hamid Badali, Abdullah M. S. Al-Hatmi1

**Affiliations:** 1 Invasive Fungi Research Center, Communicable Diseases Institute, Mazandaran University of Medical Sciences, Sari, Iran; 2 Department of Medical Mycology, School of Medicine, Mazandaran University of Medical Sciences, Sari, Iran; 3 Department of Medical Mycology and Parasitology, School of Medicine, Babol University of Medical Sciences, Babol, Iran; 4 Department of Visceral, Transplant and Thoracic Surgery, Medical University of Innsbruck, Innsbruck, Austria; 5 Department of Medical Parasitology and Mycology, School of Public Health, Tehran University of Medical Sciences, Tehran, Iran; 6 Molecular Medicine Research Center, Hormozgan Health Institute, Hormozgan University of Medical Sciences, Bandar Abbas, Iran; 7 Department of Medical Parasitology and Mycology, School of Medicine, Jahrom University of Medical Sciences, Jahrom, Iran; 8 Department of Laboratory Sciences, Sirjan School of Medical Sciences, Sirjan, Iran; 9 Medical Faculty, University of Niš, Niš, Serbia; 10 Public Health Institute of Niš, Niš, Serbia; 11 Department of Food Science and Technology, Damghan Branch, Islamic Azad University, Damghan, Iran; 12 Department of Molecular Microbiology and Immunology, South Texas Center for Emerging Infectious Diseases, The University of Texas at San Antonio, San Antonio, USA; 13 Natural and Medical Sciences Research Center, University of Nizwa, Nizwa, Oman; 14 Center of Expertise in Mycology, Radboud University Medical Center/Canisius Wilhelmina Hospital, Nijmegen, The Netherlands

**Keywords:** Diagnosis, Fungal ear infection, Fusariosis, Meta-analysis, Systematic review, Treatment

## Abstract

**Background and Purpose:**

Otomycosis caused by *Fusarium* species has been increasingly documented in recent years. This study aimed at an overview of clinical presentations, diagnostic methods, treatment alternatives, epidemiology, and future management strategies for this infection.

**Materials and Methods:**

A literature search was conducted in five scientific databases from 1966 to July 2023. The keywords included "*Fusarium*", "fusariosis", "otomycosis", "otitis externa", "ear disorder", and "ear infection". After title and abstract screening, 354 papers advanced to full-text screening; subsequently, 343 were excluded as non-relevant or case reports, leaving 11 studies to be included in this review.

**Results:**

*Fusarium* otomycosis primarily occurs in healthy individuals, particularly those with diabetes or a history of trauma or ear infections. Clinical symptoms include pruritus, pain, otorrhea, hearing loss, and external ear canal inflammation. Diagnosis mainly uses conventional methods, though molecular techniques offer accurate species identification. Treatment is challenging due to resistance to traditional antifungals; however, topical agents, like terbinafine, voriconazole, amphotericin B, and natamycin, show promise in management. In this review, the pooled prevalence of otomycosis due to *Fusarium* species is estimated at 2.3 (95% CI= 1.2-3.7).

**Conclusion:**

The findings indicated that otomycosis caused by *Fusarium* species is an emerging clinical entity that warrants attention. Considering the resistance of *Fusarium* species to most currently available antifungal drug classes, physician awareness and proper diagnostic techniques are essential for timely diagnosis, accurate identification, and appropriate management of this infection.

## Introduction

Otomycosis refers to a sub-acute or chronic inflammation and infection of the external auditory canal (EAC), which, if left untreated, can spread to the middle ear. Clinical hallmark symptoms that characterize this infection include itching, ear pain, otorrhea, pruritus, tinnitus, and hearing loss [ 1 , 2 ]. Despite the widespread distribution of otomycosis worldwide, it is more prevalent in regions with tropical and subtropical climates [ 3 ]. It is estimated to account for approximately 30% of all ear infections [ 4 ]. Otomycosis is more prevalent among individuals aged 21-40; however, conflicting results have been reported concerning its gender distribution [ 5 ]. In addition to climate characteristics, the risk factors contributing to this disease include swimming, use of hearing aids, long-term or excessive use of broad-spectrum antibiotics, anatomical abnormalities, immunodeficiency, and alterations of ear cerumen [ 6 , 7 ]. 

Although different species of yeasts and filamentous fungi can cause otomycosis, the dominant causative agents are *Aspergillus* species (i.e., *Aspergillus* section *Nigri* and *Aspergillus* section *Flavi*) and *Candida* species (i.e., *C. albicans* and *C. parapsilosis*). However, it is important to mention *Candida auris* as a multidrug-resistant emerging yeast. Since the first report of its involvement in the ear canal of a Japanese patient in 2009, *C. auris* has been isolated in over 50 countries across six continents. Its rapid transmissibility has resulted in many outbreaks worldwide, and it has been isolated from patients with otomycosis in several cases [ 8 - 11 ]. Unlikely, otomycoses rarely can develop due to dermatophytes or non-dermatophyte fungi, such as *Penicillium species*, *Fusarium* species, Mucoalean fungi, *Scopulariopsis* species, and *Alternaria* species [ 12 - 15 ]. Management and treatment of otomycosis are complicated due to the potential for recurrence of the infection and the development of drug resistance [ 4 , 16 ]. Identifying etiological agents is possible only through laboratory analyses, including mycological and bacteriological examination [ 17 ]. Effective and beneficial treatment of otomycosis involves removal of secretions and debris, and administration of appropriate local and, in rare cases, systemic antifungal agents [ 14 , 16 ], followed by management and control of predisposing factors. Although there are no universally approved treatment protocols and guidelines for otomycoses, it is worth noting that imidazole and triazole derivatives are the most commonly used antimycotic agents for local application [ 4 ]. Otomycosis is caused by *Fusarium* species is an emerging clinical entity. Since *Fusarium* often exhibits resistance to most antifungal agents, including azoles, echinocandins, and polyenes, it is crucial to consider it in the differential diagnosis to guide effective treatment and monitoring. Evidence and reports on the prevalence of *Fusarium* otomycosis, diagnostic procedures, antifungal susceptibility, treatment approaches, and disease outcomes are limited [ 18 ]. This systematic review evaluated all available reports in the relevant literature to contribute to understanding *Fusarium* otomycosis globally regarding clinical features, diagnosis, treatment, and epidemiology, which could help healthcare professionals improve patient outcomes.

## Materials and Methods

**Figure 1:**
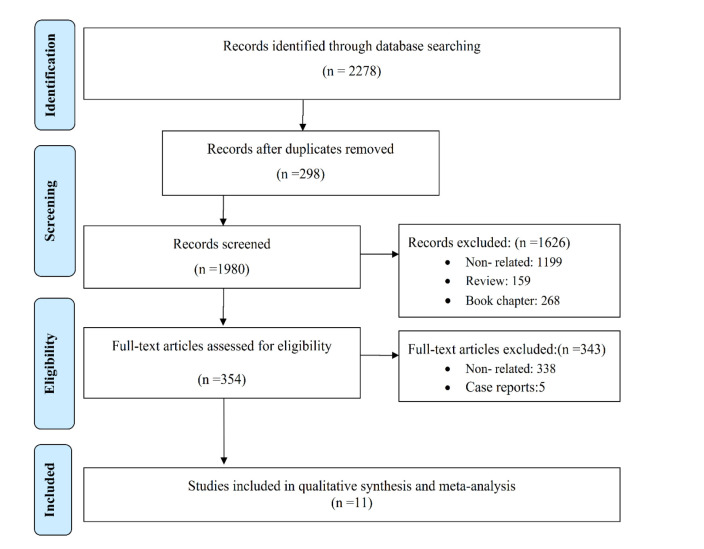
PRISMA flow diagram of the search strategy

To evaluate *Fusarium* otomycosis, 11 studies were included from a literature search of four available scientific databases, such as "PubMed," "Scopus," "ScienceDirect, and "Web of Science", and one scientific search engine, Google Scholar, from 1966 to July 2023. The keywords for literature search included the following terms: "*Fusarium*", "fusariosis", "otomycosis", "otitis externa", "ear disorder", and "ear infection". Studies were considered eligible if they met the inclusion criteria. The main inclusion criteria were otomycosis caused by *Fusarium* species. In contrast, studies were excluded from consideration if they were reviewing articles that summarized existing research or studies that only reported final results without providing access to the original data. All non-English language (except abstract) and duplicate articles were excluded from the study, and the PRISMA flow diagram was constructed (Figure 1). 

The title and abstract screening were performed independently by two reviewers (J. J. and Z. T. H.). In case of conflict between the two reviewers, a third reviewer was included to solve the conflict. All initially searched articles were imported into the EndNote software (version 20, Clarivate Analytics, USA). The statistical analysis was performed using the StatsDirect (version 3, package StatsDirect Corp, Wirral, UK). The heterogeneity index for all studies was determined using the χ2-based weight of each study, and the horizontal lines drawn along the x-axis are 95% CI values. The funnel plot (Figure S1) and Egger's regression test were performed to check publication bias.

## Results and Discussion

After the title and abstract screening, 354 relevant papers were obtained for full-paper screening. During the complete paper screening process, 343 papers were excluded as non-relevant or case reports. A data extraction Table 1 was used to report the following variables: author’s name, year of publication, study location, total otomycosis sample size, and *Fusarium* species (Table 1) [ 6 , 16 , 19 - 27 ]. 

**Table 1:** Data extraction and characteristics of included studies

No.	Author/Publication Year/Reference	Location	*Fusarium* otomycosis no./Total sample no. (%)	*Fusarium* Spp. (No.)	Diagnostic method	Treatment	Study Period	Study population
1	Bassiouny et al. 1986 [ 19 ]	Egypt	3/298 (1)	*Fusarium Oxysporum* (3)	Microscopy and culture	ND	ND	Otomycotic patients
2	Gugani et al.1989 [ 20 ]	Nigeria	1/67 (1.49)	*Fusarium* spp. (1)	Microscopy and culture	ND	ND	Clinically suspected of otomycosis
3	Kombila et al.1989 [ 21 ]	Gabon	1/83 (1.2)	*Fusarium* spp. (1)	Microscopy and culture	ND	ND	Otomycotic patients
4	Baveja et al.1993 [ 22 ]	India	1/25 (4)	*Fusarium* spp. ( 1)	Microscopy and culture	Tolnaftate.	ND	Clinically suspected of otomycosis
5	Enweani et al.1997 [ 23 ]	Nigeria	4/64 (6.25)	*F. solani* (4)	Microscopy and culture	ND	ND	Malnourished and healthy children
6	Jia et al. 2012 [ 16 ]	China	1/108 (0.92)	*F. solani* (1)	Microscopy and culture	Topical Fluconazole	Sep 2009- Sep 2010	Otomycotic patients
7	Kazemi et al. 2015 [ 24 ]	Iran	2/129 (1.55)	*Fusarium* spp. (2)	Microscopy and culture	ND	2009- 2011	Clinically suspected of otomycosis
8	Kulal et al. 2017 [ 25 ]	India	1/135 (0.74)	*Fusarium* spp. (1)	Microscopy and culture	ND	Nov 2008- Aug 2010	Clinically suspected of otomycosis
9	Salari et al. 2017 [ 26 ]	Iran	1/26 (3.84)	*Fusarium* spp. (1)	Microscopy, culture, and PCR	ND	Mar 2004- Mar 2014	Suspected of Superficial and cutaneous fungal infections
10	Alarid-Coronel et al.2018 [ 6 ]	Mexico	5/40 (12.5)	*Fusarium* spp. (5)	Microscopy and culture	Topical Antifungals	Aug 2010- Jan 2016	Immunocompetent patients
11	Kiakojuri et al. 2019 [ 27 ]	Iran	1/161 (0.62)	*Fusarium* spp. (1)	Microscopy and culture	Clotrimazole	ND	Clinically suspected of otomycosis

ND: not determined, PCR: polymerase chain reaction

### Epidemiology of Fusarium otomycosis

**Figure 2:**
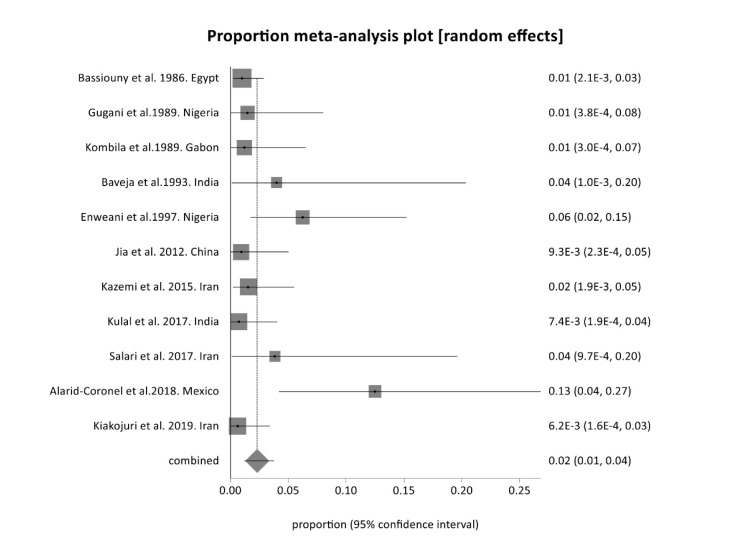
Forest plot for meta-analysis from 11 studies on *Fusarium* otomycosis

A preliminary search of five databases yielded 2278 articles and 11 meta-analysed epidemiological studies for *Fusarium* otomycosis (Figure 1). The worldwide prevalence of otomycosis is estimated at approximately 1-4%. In the present review, the pooled prevalence of otomycosis due to *Fusarium* species is estimated at 2.3 (95% CI= 1.2-3.7) (Figure 2). The heterogeneity analysis of pooled data on all included studies revealed significantly high heterogeneity (Cochran Q= 18.5 (df= 10), *p* = 0.05) and (I2 =45.9% (95% CI= 0-71.6%). The highest prevalence was reported in Mexico (13%), and the lowest prevalence was related to Iran (0.62%). Among the *Fusarium* identified in otomycosis, the most common species of *Fusarium* were *Fusarium* spp. 13/21 (76.2%), followed by *F. solani* 5/21 (23.8%) and *F. oxysporum* 3/21 (14.3) (Table 1) [ 6 , 16 , 19 - 27 ].

As the included otomycosis studies did not report gender and age distributions according to the causative fungal agent, the demographic analysis was conducted on the aggregate data. No consistent pattern was observed in terms of gender distribution, with the reported prevalence of otomycosis varying between male and female predominance across different studies [ 1 , 6 , 16 , 22 - 26 ]. This suggests that local environmental or occupational factors influence the condition rather than a fixed biological characteristic. The highest incidence rate of otomycosis was observed among young and middle-aged adults (21-40 years) [ 6 , 22 , 24 , 25 ]. While cases occur across all age groups, children and the elderly are less frequently affected. This demographic pattern may be linked to greater exposure to risk factors, such as swimming and earphone use, in younger populations.

### Risk factors

Otomycosis refers to the superficial mycotic infection of the outer ear canal associated with various predisposing factors, such as alterations in ear pH and cerumen, swimming, external ear canal trauma or instrumentation, the use of hearing aid or ear prosthesis, bacterial infection, occupational exposure to a dry, dusty environment, inadequate hygiene, cleaning the external ear canal with matchsticks, and instilling oil and earwax in the external ear canal [ 4 , 5 , 28 - 30 ]. Two of the most critical factors for otomycosis are long-term moisture exposure and the use of topical antibiotics (fluoroquinolones) or steroid eardrops [ 4 , 14 , 31 ]. In addition, researchers acknowledge physiological conditions, such as pregnancy, diabetes mellitus, malignancy, and HIV infection, as significant predisposing factors [ 3 ].

### Clinical manifestation

According to the results of the present meta-analysis, it is evident that *Fusarium* otomycosis is an uncommon disorder but clinically significant fungal infection [ 32 , 33 ]. The clinical manifestations are characterized by a broad spectrum of symptoms and signs not specific to *Fusarium* infection, resembling infections of the EAC caused by various agents. Individuals suffering from this condition typically experience symptoms, such as otalgia, otorrhea, and hearing impairment, which can progress if left untreated [ 34 ]. These initial symptoms can be followed by severe otalgia, frequently accompanied by pruritus, aural fullness, and discomfort. The presence of ear discharge, usually serous or serosanguinous, with a strong odour and debris build-up within the ear canal, can contribute to varying degrees of hearing loss, ranging from mild to severe infections [ 16 , 35 ]. Spread of the infection, especially if there is an extension to the tympanic membrane, can lead to sensorineural hearing loss if the middle ear is involved [ 14 ].

In complications, patients may experience less common symptoms, such as vertigo, tinnitus, and facial musculature weakness [ 36 ]. Physical examination in *Fusarium* otomycosis usually reveals erythematous and edematous ear canal skin, often accompanied by erosions and ulcerations [ 16 ]. In severe cases, mainly in immunocompromised patients, direct extension into adjacent structures, such as the temporomandibular joint or cranial nerves, may lead to further neurologic sequelae [ 37 ]. In differential diagnosis, it is essential to distinguish between this fungal infection and the other possible causes, such as acute otitis externa or chronic suppurative otitis media [ 2 ]. Moreover, early detection and identification of causative agents are crucial for the establishment of appropriate therapy, which can help prevent potential complications, including disease progression and associated morbidity [ 38 ].

### Diagnostic approaches

*Fusarium* otomycosis is a relatively uncommon but increasingly recognized clinical entity that poses a diagnostic challenge due to its symptom similarity to other forms of ear infections, as well as the absence of standardized laboratory examinations. The initial steps in diagnosing approaches are gathering anamnestic data on symptoms and potential risk factors, followed by clinical examination-otoscopy. Knowledge of the ecological and epidemiological characteristics of the particular region is also essential in diagnosing this superficial fungal infection. During the clinical examination, healthcare professionals generally cannot differentiate *Fusarium* otomycosis from infection of a different etiology based solely on the observed symptoms and clinical signs [ 35 ]. Therefore, a laboratory diagnosis is necessary to manage this condition. If feasible, various diagnostic modalities are available, including conventional mycological techniques and molecular methods. Given that yeast and molds can be members of the microbiota naturally present on the skin of the EAC or represent fungal transient flora, the standard approach to address this dilemma is to perform multiple successive analyses using several samples, and in sporadic cases, confirmation can be achieved through histopathology [ 15 ]. 

Conventional mycological methods include microscopic examinations, which allow detecting fungal elements in samples but may not establish the specific etiological agents. On the other hand, cultivation represents the gold standard for isolation and identification of *Fusarium* species from clinical specimens. This culture-based mycological diagnosis includes inoculation of clinical specimens, such as ear discharge or tissue, onto appropriate fungal culture media (i.e., sabouraud dextrose agar and malt extract agar). The colonies that appear after 2-3 days of incubation at 25-30° care subsequently examined macroscopically and microscopically to identify the genus or species of fungi [ 2 ]. Molecular-based identification and matrix-assisted laser desorption/ionization-time of flight-mass spectrometry can be promising and robust tools to identify *Fusarium* species at the species level [ 39 ].

The DNA sequencing of the translation elongation factor 1α (*TEF1α*) gene has recently been developed to improve the sensitivity and specificity of the laboratory identification of *Fusarium* species [ 40 ]. Sequencing DNA and determining molecular targets for identification is an approach that could enable the design and establishment of rapid molecular testing for otomycosis [ 41 ]. Imaging studies, such as computed tomography or magnetic resonance imaging, may be used to determine the extent of the infection and to identify any underlying conditions that may be contributing to the infection [ 42 ]. It is important to note that the laboratory diagnosis of otomycosis due to *Fusarium* species can be challenging, and a combination of methods may be needed to confirm the diagnosis, since identification of *Fusarium* species is crucial for appropriate treatment.

### Treatment options

Treatment of otomycosis is currently a big concern and challenge for otolaryngologists and physicians [ 7 ]. This is mainly due to the lack of established guidelines for diagnosis and treatment, and undefined therapy duration [ 15 , 43 , 44 ]. Considering their remarkably high prevalence, most infections of the external auditory canal are treated without reliance on laboratory-based evidence. Furthermore, inadequate laboratory practices, such as the omission of mycological examinations, as well as the fact that in some instances, mycological examination does not include the procedure for isolation of all potential causative fungi (contamination), make the treatment of otomycoses, including fusariosis, challenging. The issue becomes more serious when considering the potential resistance to currently available antifungal agents [ 7 ]. Dealing with infections caused by *Fusarium* species can be quite daunting, as clinically relevant *Fusarium* species are often found to be resistant to most antifungal agents, including azoles, echinocandins, and polyenes [ 18 ]. Additionally, it is worth emphasizing that *Fusarium* species exhibit intrinsic resistance to most currently available antifungal drug classes, such as azoles and echinocandins, even without prior exposure to these agents [ 18 , 45 ]. 

Findings of the present study and published data indicate that the successful treatment of otomycosis caused by *Fusarium* species is rare. Nevertheless, earlier studies have suggested that terbinafine could be a potential candidate for specific types of superficial infections caused by *Fusarium* species, supported by the results of a recent study conducted by Ting-Hua Yang, which demonstrated the non-toxic nature of this antimycotic to the inner ear end organs at a dosage of 0.4 mg [ 15 , 18 , 46 - 49 ]. Natamycin (5%), topical amphotericin B (0.5%), and 1% topical voriconazole for up to weeks are successfully employed to treat superficial *Fusarium* infections [ 50 , 51 ]. Echinocandins exhibit limited effectiveness against *Fusarium* species and are generally ineffective in treating infections caused by *Fusarium* species [ 18 ]. Combining antifungal agents has improved treatment efficacy in cases where mono-antifungal therapy proves ineffective [ 18 ]. Several *in vitro* or *in vivo* studies using an experimental murine model have explored the potential synergetic effect of combining various antifungal agents [ 52 - 54 ]. Terbinafine and voriconazole have exhibited a synergistic effect against different species of *Fusarium*; additionally, caspofungin, while demonstrating high minimum effective concentrations (MECs) against several *Fusarium* species when used alone, has shown a notably strong synergistic effect when combined with other antifungals [ 55 ]. In an experimental murine model, combination therapy of terbinafine and liposomal amphotericin B has shown promising results in treating *Fusarium* infections. Other antifungal agents, such as posaconazole or voriconazole in combination with amphotericin B, have displayed poor effectiveness against these species [ 56 , 57 ]. Al-Hatmi et al. found that a combination of natamycin and voriconazole demonstrated 70% *in vitro* synergistic interactions against a considerable portion of *Fusarium* isolates [ 58 ]. According to Spader et al., *in vitro* experiments revealed synergistic effects when amphotericin B was combined with rifampin, 5-flucytosine, caspofungin, and voriconazole [ 59 ]. Nosratabadi et al showed that luliconazole, lanoconazole, and efinaconazole had good activity against all *Fusarium* isolates, the minimum inhibitory concentration of luliconazole, lanoconazole and efinaconazole were in the ranges of 0.001–0.125, 0.001–0.5, and 0.064–4 μg/ml, respectively, and 272 of all isolates (96.4%) were inhibited in the concentration of ≤0.125 μg/ml of luliconazole and lanoconazole [ 60 ]. Nosratabadi et al. investigated the in vitro antifungal susceptibility pattern of miltefosine against a collection of azole and echinocandin-resistant *Fusarium* strains. Their results revealed that amphotericin B (0.8µg/mL) had the lowest geometric mean MICs/MECs values, followed by miltefosine (1.44µg/mL), voriconazole (2.15µg/mL), caspofungin (7.23µg/mL), and itraconazole (14.19µg/mL). Therefore, after amphotericin B, miltefosine has shown superior efficacy against *Fusarium* isolates, compared to azoles and echinocandins. This suggests its potential as a novel treatment for *Fusarium* infections and warrants further investigation through *in vivo* efficacy studies [ 61 ]. More clinical studies are needed to examine the most effective combination for the treatment of *Fusarium* infections, and additional research is recommended to address this issue [ 18 ].

## Conclusion

Otitis externa is an infection of the ear canal caused by bacteria and fungi (otomycosis), which can cause pain due to inflammation. If otitis externa does not respond to treatment and involves the bony structures, it can progress to malignant otitis externa. This infection can spread from the outer ear to nearby tissues and the temporal bone through the fissure of Santorini. Findings of the present study, together with published data, indicate that otomycosis caused by *Fusarium* species is an emerging clinical entity that warrants attention. Awareness among physicians and the use of appropriate diagnostic techniques are essential for the timely identification and effective management of infections. Considering the potential complications, such as direct extension into joint or cranial nerves, as well as the resistance of *Fusarium* species to regular antifungal agents with a different mechanism of action, it is crucial to include otomycosis caused by non-dermatophyte fungi in the differential diagnosis, followed by effective treatment and monitoring.
